# Exploring the Mechanism of Action Compound-Xueshuantong Capsule in Diabetic Retinopathy Treatment Based on Network Pharmacology

**DOI:** 10.1155/2020/8467046

**Published:** 2020-09-09

**Authors:** Haoran Li, Biao Li, Yanlin Zheng

**Affiliations:** Hospital of Chengdu University of Traditional Chinese Medicine, Chengdu 610072, China

## Abstract

**Materials and Methods:**

The components with oral bioavailability ≥30% and drug similarity ≥0.18 were screened by the Traditional Chinese Medicine System Pharmacology Database and Analysis Platform (TCMSP), and the effective grouping of Compound-Xueshuantong Capsule was obtained. At the same time, the targets of each drug active component in the Compound-Xueshuantong Capsule were obtained by searching the TCMSP. The effective components and targets of the Compound-Xueshuantong Capsule were annotated by the UniProt database, and the disease treatment targets were searched by the GeneCards database. The disease treatment target is intersected with the drug target and the Wayne diagram is drawn by VennDiagram. The active ingredient targets of the intersection and Compound-Xueshuantong Capsule were inputted into Cytoscape 3.7.2 software to construct the active ingredient-target-disease interaction network. The above targets were inputted into the String database for protein-protein interaction network prediction. Finally, by using the DAVID database, GO and KEGG enrichment analysis was carried out to reveal the potential signal pathway of the Compound-Xueshuantong Capsule in diabetic retinopathy treatment.

**Results:**

93 active components of the Compound-Xueshuantong Capsule and 92 targets for treating diabetic retinopathy were screened. The main active components of the Compound-Xueshuantong Capsule in treating diabetic retinopathy were quercetin, luteolin, kaempferol, beta-sitosterol, isorhamnetin, and tanshinone IIa. The effect of the Compound-Xueshuantong Capsule on diabetic retinopathy may be related to IL6, EFGR, CASP3, and VEGFA. In addition, the treatment of diabetic retinopathy mainly involves in the regulation of nuclear receptors and transcription factors in vivo. The target of the Compound-Xueshuantong Capsule in diabetic retinopathy treatment is significantly enriched in the AGE-RAGE signal pathway, TNF signal pathway, HIF-1 signal pathway, and VEGF signal pathway in diabetic complications.

**Conclusion:**

Compound-Xueshuantong Capsule can treat diabetic retinopathy through multitarget, multipathway, and multipathway regulation of the biomolecular network. The potential biological mechanism of the Compound-Xueshuantong Capsule in diabetic retinopathy treatment may be related to the AGE-RAGE signal pathway, TNF signal pathway, HIF-1 signal pathway, and VEGF signal pathway in diabetic complications, but these findings still need to be confirmed by further clinical research.

## 1. Introduction

Diabetes is a disease that affects the organs and the blood glucose metabolism of all organs throughout the body. It is mainly divided into type 1 and type 2 diabetes, which can cause microvascular diseases such as brain, kidney, heart, and retina. Diabetic Retinopathy (DR) is one of the most serious microvascular complications and a disease with a high incidence of blinding eye disease. In 2015, there were an estimated 36 million blind and 216 million visually impaired DR patients in the world, mainly in the Asia-pacific region, which shows its harm to the population [1].

The pathogenesis of DR is complex, and hyperglycemia is recognized as the initiating factor. On the one hand, hyperglycemia can damage retinal capillaries through a variety of metabolic pathways; on the other hand, it can induce apoptosis of retinal pericytes, leading to occlusion, ischemia, and leakage of capillaries [2]. Recent studies have shown that chronic inflammation and neurodegeneration may further aggravate retinal ischemia and hypoxia [3]. Chronic ischemia and hypoxia, which lead to retinal punctured hemorrhage, microaneurysm, and hard exclusion, are the main pathological features of NPDR. At the same time, the activated renin-angiotensin system (RAS) [2] and vascular endothelial growth factor (VEGF) [3] accelerated the deterioration of NPDR by promoting the proliferation of endothelial cells and new blood vessels, which eventually led to the progression of NPDR to PDR.

Currently, the methods widely used in the treatment of diabetic retinopathy include retinal steroid drugs, antivascular endothelial growth factor (VEGF) drugs, laser photocoagulation, and vitrectomy. The current treatment is mainly aimed at the advanced stage of proliferative diabetic retinopathy, and the effect is limited. Therefore, the discovery of new effective drugs and the formulation of effective prevention and treatment measures are of great clinical significance for the prevention and control of the occurrence and development of DR, so as to improve the prognosis of patients.

Compound-Xueshuantong Capsule (Z20030017) is a compound capsule made up of four traditional Chinese medicines: SanQi (*Panax notoginseng* (Burkill) Chen (Araliaceae)), HuangQi (*Astragalus membranaceus* (Fisch.) Bunge (Leguminosae)), DanShen (*Salvia miltiorrhiza* Bunge (Lamiaceae)), and XueSheng (*Scrophularia ningpoensis* Hemsl (Scrophulariaceae)) in the ratio of 25 : 8 : 5 : 8. It has been proved to have a good clinical effect in the treatment of diabetic retinopathy. In a clinical trial of 28 patients (30 eyes) from China, the total effective rate of Compound-Xueshuantong Capsule in improving the vision of diabetic retinopathy was 76.6%, and the improvement rate of fundus hemorrhage was 70% [4]. Meanwhile, in another clinical trial of 54 patients (68 eyes) from China, the total effective rate of treatment with Compound-Xueshuantong Capsule was 91% (31/34), which was significantly higher than that of the control group (68% (23/34), and the difference was statistically significant (*p* < 0.05) [5]. At the same time, studies have also shown [6, 7] that Compound-Xueshuantong Capsule has a significant therapeutic effect on STZ-induced diabetic retinopathy in rats. All the above studies show that Compound-Xueshuantong Capsule is an effective drug for the clinical diabetic retinopathy treatment.

Compound-Xueshuantong Capsule has been proved to reduce STZ-induced retinal injury in rats. In addition, Compound-Xueshuantong Capsule can inhibit AR activity and correct the overexpression of retinal VEGF, ICAM-1, and ET-1 and the low expression of PEDF [7]. Since the pharmacological mechanism of the Compound-Xueshuantong Capsule and its active components is still unclear, finding new strategies to identify the therapeutic targets that exist in DR and active compounds is of great significance.

Network pharmacology is a new discipline, which can clarify the role of traditional Chinese medicine by constructing a disease-target-drug network and screening out special nodes [8]. Using network pharmacology, this study investigated the potential biological mechanism of the Compound-Xueshuantong Capsule in diabetic retinopathy treatment.

## 2. Materials and Methods

### 2.1. Standardization of Chinese Medicine Names in Compound-Xueshuantong Capsule

We used “The Plant List” (http://www.theplantlist.org) and related literature [7] to retrieve the complete and standardized names of the four traditional Chinese medicines in Compound-Xueshuantong Capsule, in which the complete name of SanQi is “*Panax notoginseng* (Burkill) Hen (Araliaceae)”. The complete name of HuangQi was *Astragalus membranaceus* (Fisch.) Bunge (Leguminosae), DanShen was Salvia miltiorrhiza Bunge (Lamiaceae), and XuanShen was *Scrophularia ningpoensis* Hemsl (Scrophulariaceae). For the convenience of expression, we use the local names of the four traditional Chinese medicines as follows: SanQi, HuangQi, DanShen, and XuanShen.

### 2.2. Collection and Screening of Active Components

The active components of SanQi, HuangQi, DanShen, and XuanSheng in Compound-Xueshuantong Capsule were searched in the TCMSP database (http://tcmspw.com/index.php). The active components of the Compound-Xueshuantong Capsule were obtained by using oral bioavailability (OB) ≥30% and drug-like (DL) ≥0.18 as screening conditions [9] (OB represents the oral availability of drug components, and DL refers to the similarity between ingredients and known drugs).

### 2.3. Collection and Screening of Potential Targets of Active Components

Based on the TCMSP database [9], the active components of the Compound-Xueshuantong Capsule were matched with potential targets one by one, and the database UniProt (http://www.uniport.org) was used to retrieve targets from different sources and obtain official gene symbols of all target genes for subsequent analysis.

### 2.4. Screening of Disease Treatment Targets

In the GeneCards database (https://www.genecards.org), diabetic retinopathy was used as the keyword to search the disease treatment targets of diabetic retinopathy.

### 2.5. Construction of Drug-Disease Target Network

In order to clarify the relationship between the drug Compound-Xueshuantong Capsule and the disease treatment target, the drug potential targets were intersected with the disease treatment targets, and they were used as the important targets for Compound-Xueshuantong Capsule in diabetic retinopathy treatment. We get the construction of an active component-target-disease-drug interaction network by using Cytoscape 3.7.2, an open-source bioinformatics software platform for visualizing molecular interaction networks [10]. In the constructed network, nodes are used to represent active components and targets, and edges are used to represent the effective relationship between them. In addition, Network Analyzer in Cytoscape software is used to calculate topological parameters such as Degree to evaluate the importance of active components and targets.

### 2.6. Protein-Protein Interaction Network (PPI) Construction for Diabetic Retinopathy Treatment

The important targets of the Compound-Xueshuantong Capsule for diabetic retinopathy treatment were input into the String database (https://string-db.org), and the study species was limited to “*Homo sapiens*” for protein-protein interaction network (PPI) prediction. The correlation degree of target proteins in the PPI network was calculated to screen out the top 30 most densely correlated target proteins, and the histogram was calculated.

### 2.7. Functional Enrichment Analysis of GO and Analysis of KEGG Signal Pathway

By using the DAVID 6.8 database [11], we analyze the GO function enrichment and KEGG signal pathway enrichment of the important targets of the Compound-Xueshuantong Capsule in diabetic retinopathy treatment.

## 3. Result

### 3.1. Active Components of Compound-Xueshuantong Capsule

By searching the TCMSP database, 119 chemical constituents of SanQi, 87 chemical constituents of HuangQi, 202 chemical constituents of DanShen, and 47 chemical constituents of XuanShen were obtained. There are 9 active components of XuanShen, 65 active components of DanShen, 20 active components of HuangQi, and 8 active components of SanQi.

### 3.2. Collection and Screening of Active Components of Compound-Xueshuantong Capsule

We predict the targets of the above active ingredients by using the TCMSP database, and 113 related targets were screened out. The targets from different sources were annotated with the UniProt (http://www.uniport.org) database for subsequent analysis.

### 3.3. Screening of Disease-Related Targets

Diabetic retinopathy was used as the keyword to search the disease-related targets of diabetic retinopathy in the GeneCards database (https://www.genecards.org). 2549 disease targets related to diabetic retinopathy were obtained.

### 3.4. Construction of Drug-Disease Target Network

**Figure 1:**
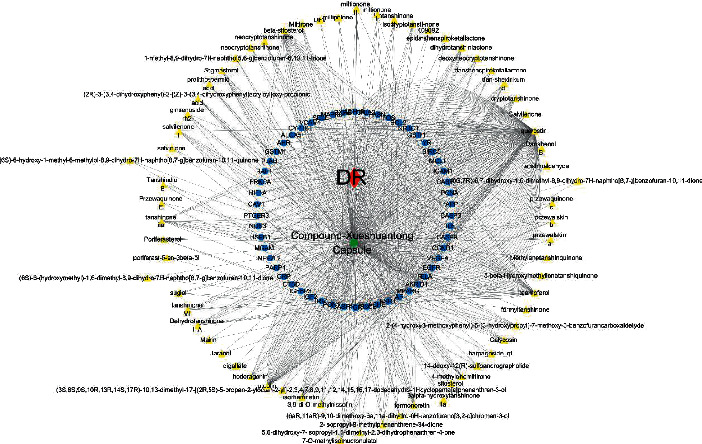
Active component-target network of Compound-Xueshuantong Capsule in diabetic retinopathy treatment.

92 drug component-disease common targets were obtained by intersecting the above 2549 disease targets and 113 drug component targets, which were used as the therapeutic targets of the Compound-Xueshuantong Capsule in diabetic retinopathy treatment. The collected active components and drug components-disease common targets of Compound-Xueshuantong Capsule were inputted into Cytoscape 3.7.2 software, and network visualization and topological analysis were carried out (Figure 1). The network consists of 1160 nodes (66 active component nodes, 1 disease node, 1 drug node, and 54 target nodes) and 1160 edges, as shown in Figure 1. The Degree and the Betweenness Centrality of the node are calculated by using Network Analyzer in the Cytoscape software. The Degree and the Betweenness Centrality are the centrality indicators of the network nodes. The larger the Degree and the Betweenness Centrality are, the higher the centrality of the nodes is and the more important it is in the network. On this basis, we find the node degrees of the top ten active ingredients (Table 1). They are quercetin, luteolin, kaempferol, beta-sitosterol, isorhamnetin, tanshinone IIa, cryptotanshinone, formononetin, Dehydrotanshinone II A, and dan-shexinkum d. Therefore, these active ingredients are closely related to the target and may be the key components in the treatment of diabetic retinopathy in the Compound-Xueshuantong Capsule.

### 3.5. Construction of Protein-Protein Interaction Network (PPI) for the Therapeutic Target of Diabetic Retinopathy

**Figure 2:**
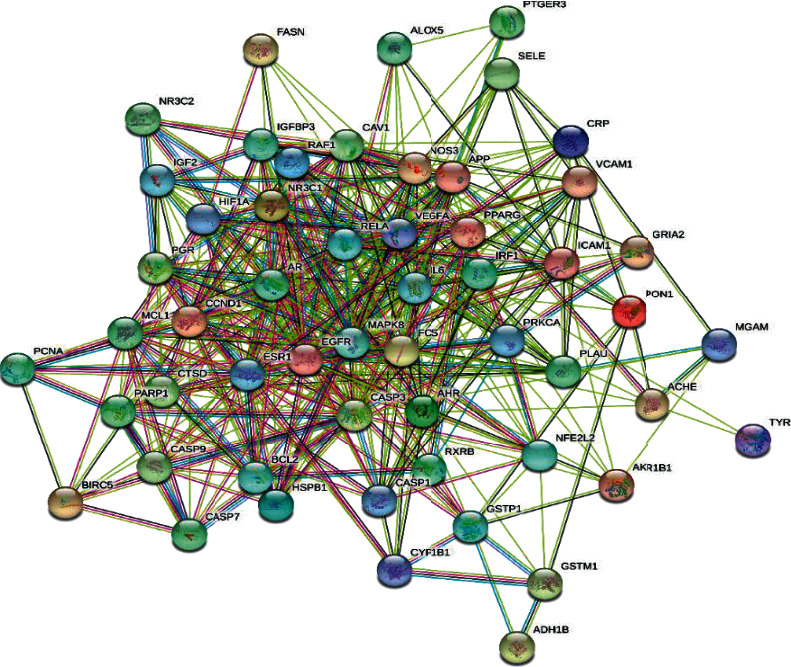
Protein-protein interaction (PPI) network of Compound-Xueshuantong Capsule in diabetic retinopathy treatment.

**Figure 3:**
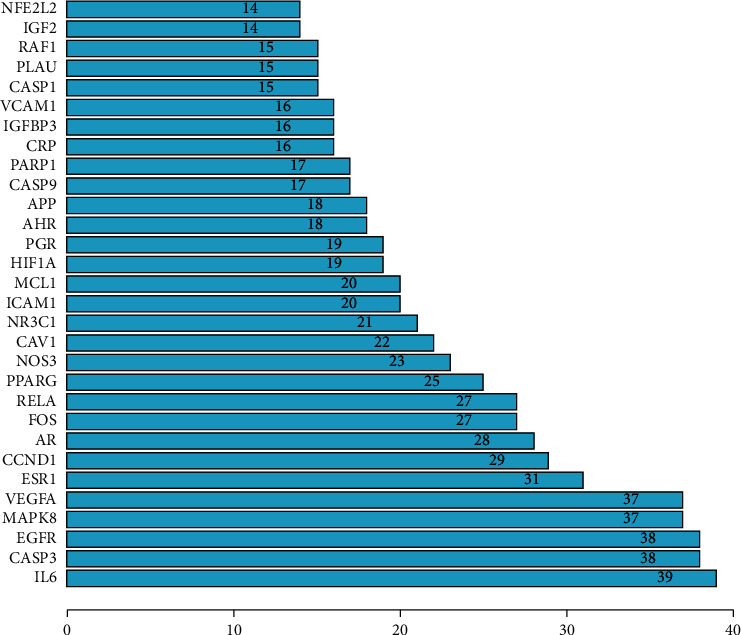
Core targets of Compound-Xueshuantong Capsule in diabetic retinopathy treatment.

The important targets of Compound-Xueshuantong Capsule for diabetic retinopathy treatment were input into the String database (https://string-db.org), the study species was limited to “*Homo sapiens*,” the protein-protein interaction network (PPI) was predicted, and then the PPI network was obtained (Figure 2). A total of 54 targets can interact with proteins, and 436 edges represent the interactions between proteins. EXCEL is used to calculate the value of the edge between nodes. The larger the value of the edge is, the more the interaction is, which plays a more core role in the PPI network. The histogram was drawn as shown in Figure 3. The top 30 targets were IL6, CASP3, EGFR, MAPK8, VEGFA, ESR1, CCND1, AR, FOS, RELA, PPARG, NOS3, CAV1, NR3C1, ICAM1, MCL1, HIF1A, PGR, AHR, APP, CASP9, PARP1, CRP, IGFBP3, VCAM1, CASP1, PLAU, RAF1, IGF2, and NFE2L2.

### 3.6. Results of GO Functional Enrichment Analysis

**Figure 4:**
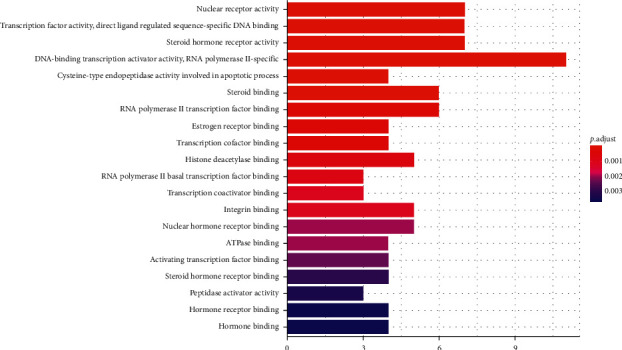
Histogram of GO functional enrichment analysis.

**Figure 5:**
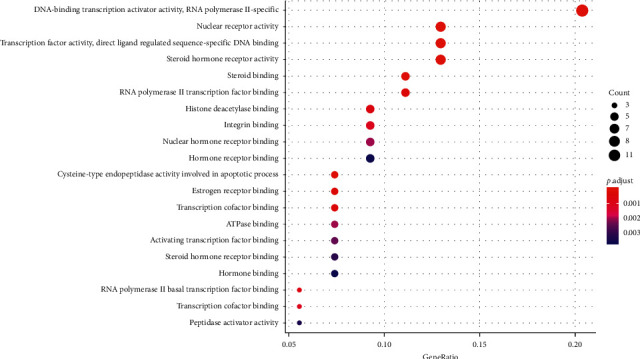
Bubble diagram of GO functional enrichment analysis.

We input 70 drug composition-disease common targets into the DAVID 6.8 database [11] for GO function enrichment analysis (*p* < 0.05, *q* < 0.05), from which a total of 62 GO items are obtained. From these items, the functional information of the top 20 (from small to large) with *p* value is selected, and the bubble chart and histogram are drawn with *R* version 3.6.1, as shown in Figures 4 and 5. The y-coordinate in Figure 4 represents the GO entry. The abscissa represents the number of genes enriched on GO items, and the color represents the size of *p* value. The redder the color, the smaller the *p* value and the higher the degree of enrichment. The y-coordinate in Figure 5 represents the GO entry. The abscissa is the ratio of the number of genes enriched on the GO entry to the total target genes. The color represents the size of the *p* value. The bubble size represents the number of genes enriched on the modified GO entry. Therefore, it can be seen that the biological processes involved in the treatment of diabetic retinopathy with Compound-Xueshuantong Capsule (Table 2) are mainly as follows: nuclear receptor activity, transcription factor activity, direct ligand regulated sequence-specific DNA binding, steroid hormone receptor activity, DNA-binding transcription activator activity, RNA polymerase II-specific, cysteine-type endopeptidase activity involved in the apoptotic process, steroid binding, RNA polymerase II transcription factor binding, estrogen receptor binding, transcription cofactor binding, histone deacetylase binding, RNA polymerase II basal transcription factor binding, transcription coactivator binding, integrin binding, nuclear hormone receptor binding, ATPase binding, activating transcription factor binding, steroid hormone receptor binding, peptidase activator activity, and hormone receptor binding hormone binding.

### 3.7. Enrichment Analysis of KEGG Pathway

**Figure 6:**
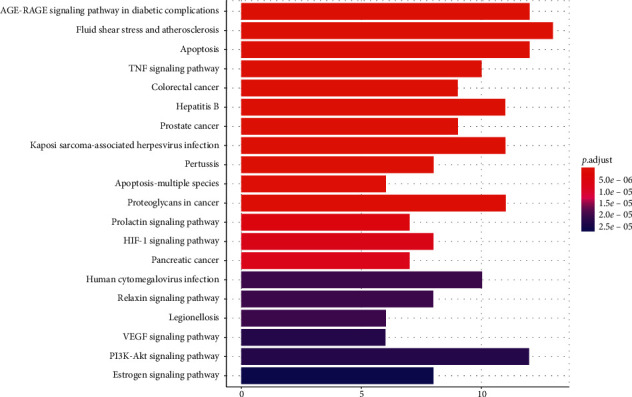
Histogram of enrichment analysis of the KEGG pathway.

**Figure 7:**
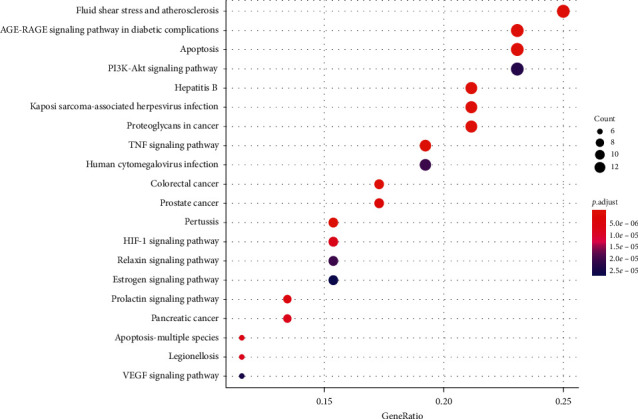
Bubble diagram of KEGG pathway enrichment analysis.

We used the DAVID6.8 database [11] for KEGG pathway enrichment analysis of drug-disease intersection genes (*p* < 0.05, *q* < 0.05), thus obtaining a total of 115 signaling pathways, from which we selected the top 20 (from small to large) signaling pathways with *p* values, and drew bubble plots and bar plots with *R* version 3.6.1, as shown in Figures 6 and 7. We used the table to list the pathway data related to target enrichment of Compound-Xueshuantong Capsule for diabetic retinopathy treatment, as shown in Table 3.

## 4. Discussion

Based on network pharmacology, we analyzed the key components and targets of Compound-Xueshuantong Capsule in diabetic retinopathy treatment by using database and software and build a network to analyze the function and pathway of the target, so as to explore the mechanism of Compound-Xueshuantong Capsule in diabetic retinopathy treatment.

Studies have shown that quercetin, luteolin, kaempferol, *β*-sitosterol, isorhamnetin, tanshinone IIa, cryptotanshinone, formononetin, Dehydrotanshinone II A, and dan-shexinkum d in Compound-Xueshuantong Capsule are the key components in the treatment of diabetic retinopathy. Among them, quercetin comes from HuangQi and SanQi. Previous studies have shown that quercetin has the effect of antiangiogenesis and may be able to combat neovascularization caused by diabetic retinopathy [12]. Other studies have shown that quercetin can reduce retinal edema, vacuoles, and other pathological changes caused by diabetes [13]. And quercetin can also control the related symptoms of diabetic retinopathy by inhibiting inflammation and oxidative stress [14, 15]. Luteolin is derived from DanShen. Studies have shown that luteolin inhibits the angiogenesis of RF/6A cells induced by vascular endothelial growth factor [16]. Therefore, luteolin may reduce the clinical manifestations of diabetic retinopathy by inhibiting angiogenesis. Kaempferol comes from HuangQi. Studies have confirmed that kaempferol can protect retinal ganglion cells from high glucose injury through ERK and VASH1 signaling pathways [17], and kaempferol can also protect human RPE cells from oxidative stress injury through its antioxidant activity and antiapoptosis function [18]. Other studies have shown that kaempferol inhibits the activation of the Src-Akt1-ERK1/2 signal pathway by targeting VEGF and PGF and thus inhibits the angiogenesis of human retinal endothelial cells [19], which may permit the treatment of neovascularization caused by diabetic retinopathy. Studies have shown that *β*-sitosterol from SanQi and HuangQi has certain blood glucose control and antioxidant effects [20, 21]. Therefore, the clinical manifestations caused by diabetic retinopathy can be improved by normalizing the changes in blood glucose and oxidative stress. Isorhamnetin comes from HuangQi. Studies have proved that isorhamnetin can protect human RPE cells from oxidative stress-induced cell death, which is related to the activation of the PI3K/Akt signal pathway [22], so it can prevent the imbalance of oxidative stress caused by diabetic retinopathy. Other studies have shown that isorhamnetin can significantly reduce pain, blood sugar levels, and anti-inflammatory effects [23]. These also help to improve the pathological changes caused by diabetic retinopathy. Tanshinone IIA comes from DanShen. Studies have shown that tanshinone IIA can improve the mitochondrial dysfunction and mitosis induced by advanced glycation end products by increasing the level of GLO1 [24], thus improving the damage of retinal endothelial cells caused by diabetic retinopathy. Other studies have shown that tanshinone IIA can inhibit the proliferation, migration, and vascularization of human retinal endothelial cells induced by high glucose, which may be related to its effect on the expression of VEGF and ICAM-1 [25].

Through the construction of the protein-protein interaction (PPI) network, we obtained the core targets of Compound-Xueshuantong Capsule in diabetic retinopathy treatment, such as IL6, EFGR, CASP3, and VEGFA.

IL-6 is one of the important interleukins in inflammatory reaction and a series of pathophysiological processes in vivo. Under the condition of diabetic retinopathy, due to the increase of mitochondrial reactive oxygen species caused by hyperglycemia, the expression of cell adhesion molecules (ICAM-1, VCAM-1) is upregulated under the action of interleukin (IL-1, IL-6) induced by reactive oxygen species, and leukocyte adhesion damages endothelial cells. At the same time, research from Zhou et al. showed that IL-6 and other inflammatory cytokines increased in the vitreous of patients with PDR, which suggested that inflammatory cytokines were closely related to the occurrence and development of PDR [26]. Research from Ran et al. showed that the secretion of inflammatory factors such as IL-6 in retinal pigment epithelial cells treated with high glucose increased [27].

EGFR is the specific receptor of EGF. After the specific binding of PDR, it plays a certain role in the formation and development of PDR in the form of autocrine or paracrine. Research from Ju et al. pointed out that EGFR in the DR model seriously promoted retinal dysfunction, damage of retinal structure and mitochondrial structure in retinal vessels and retinal vascular abnormalities (including neovascularization) in diabetic mice [28].

CASP3 (Caspase3) is an apoptosis-related gene. Research from Zhang et al. pointed out that the expression of apoptosis-related genes changed and the expression of CASP3 increased significantly in diabetic rats after drug treatment [29]. It is suggested that the induction of apoptosis may improve DR-related symptoms.

Pathological neovascularization is an important clinical manifestation of DR. VEGFA is an angiogenesis-related gene. Research from Xu et al. pointed out that the upregulation of VEGFA in retinal capillary endothelial cells and pericytes of diabetic rats can promote the occurrence and development of diabetic retinopathy [30]. Research from Yu et al. and other studies also showed that the expression of VEGFA increased in retinal microvascular endothelial cells stimulated by high glucose [31].

Further analysis of the GO functional enrichment of Compound-Xueshuantong Capsule in diabetic retinopathy treatment showed that there was a high correlation between Compound-Xueshuantong Capsule and biological processes such as nuclear receptor activity, transcription factor activity, and sequence-specific DNA binding regulated by direct ligand.

The nuclear receptor is a kind of transcriptional regulator activated by ligand. In recent years, the nuclear receptor family has received extensive attention in the field of metabolic diseases (such as diabetes, fatty liver). Some studies have pointed out that peroxisome proliferator-activated receptor *γ* (PPAR *γ*), as a member of the nuclear receptor superfamily, has significant effects on antiangiogenesis, antifibrosis, anti-inflammation, and control of oxidative stress in various organs [32, 33].

A large number of studies [34–37] have shown that the activity of transcription factors in retinal cells in high glucose environment is affected, and abnormal changes can occur in the processes of proliferation, apoptosis, oxidative stress, inflammation, and angiogenesis. To some extent, the clinical symptoms of DR can be improved by regulating the activity of transcription factors and affecting related pathways.

In the results of KEGG pathway enrichment analysis of the targets of Compound-Xueshuantong Capsule in the treatment of diabetic retinopathy, the AGE-RAGE signal pathway in diabetic complications, TNF signal pathway, HIF-1 signal pathway, and VEGF signal pathway are closely related to diabetic retinopathy.

Advanced glycation end products (AGEs) accumulate heavily in diabetes, especially in retinal vessels and epithelial cells, and have been proved to be related to diabetic retinopathy (DR). The interaction between AGEs and its receptor RAGE is necessary to induce oxidative stress and mitochondrial dysfunction [38]. Studies [39–41] have shown that some drugs can protect diabetic retinal vascular cells from injury by inhibiting the accumulation of advanced glycation end products (AGEs) and reducing the binding activity of AGEs/RAGE (AGEs receptor).

TNF (Tumor Necrosis Factor) is a kind of cytokine which can directly kill tumor cells but has no obvious toxicity to normal cells. It can be divided into TNF-*α* and TNF-*β*. Studies have shown that the secretion of inflammation-related cytokines (TNF-*α*, IL-6, etc.) in human retinal endothelial cells (HRECs) in high glucose environment increases [42], suggesting that TNF- *α* may be involved in the inflammatory response of retinal endothelial cells in high glucose environment, thus promoting the related clinical manifestations of DR. Studies have shown that after treatment, the expression level of TNF-*α* in DR model decreased [43–45]. This further confirms the important role of TNF-*α* in the pathological changes of DR.

Vascular endothelial growth factor (VEGF), as a highly specific vascular endothelial growth factor, can promote vascular endothelial cell migration, proliferation, and angiogenesis. Studies have shown that the level of VEGF in the vitreous of patients with DR is significantly increased, which greatly promotes the formation of pathological neovascularization [46].

Hypoxia-inducible factor-1 (HIF-1) is a necessary transcriptional activator that mediates cell adaptation to hypoxia. HIF is composed of HIF- *α* and HIF-*β* subunits, in which HIF- *α* is divided into HIF-1*α*, HIF-2*α*, and HIF-3*α* subtypes. There are many studies on HIF-1*α*. The main physiological function of HIF-*α* is to upregulate its expression under hypoxia so as to maintain the stability of systemic, local, and intracellular oxygen concentration. Studies have confirmed that angiogenesis plays an important role in diabetic retinopathy, and hypoxia-inducible factor-1*α* is an important transcriptional activator of vascular endothelial growth factor [47]. HIF antagonist has a significant inhibitory effect on ocular neovascularization [48], which also proves the important role of HIF-1 in pathological neovascularization.

However, in this study, we were unable to conclude that the effects of the Compound-Xueshuantong Capsule on these pathways were specifically inhibition or activation. Instead, we could only infer that the active ingredients contained in Compound-Xueshuantong Capsule had inhibitory effects on the AGE-RAGE signal pathway in diabetic complications, TNF signaling pathway, HIF-1 signaling pathway, and VEGF signaling pathway in diabetic retinopathy due to their effects on neovascularization, retinal edema, inflammatory response, and oxidative stress injury. The specific regulation process of the pathway is the next step of our team's research.

## 5. Conclusion

To sum up, this study analyzed the mechanism of action Compound-Xueshuantong Capsule in diabetic retinopathy treatment from the aspects of action target and pathway by means of TCMSP, Drugbank, STRING, Cytoscape3.7.2, and so on. After consulting the relevant research literature, combined with the analysis of the results of this study, Compound-Xueshuantong Capsule mainly targets IL6, EFGR, CASP3, and VEGFA and mainly participates in the regulation of nuclear receptor activity and transcription factor activity. Diabetic retinopathy is treated by regulating the AGE-RAGE signal pathway in diabetic complications, TNF signal pathway, HIF-1 signal pathway, and VEGF signal pathway. As shown in our study, the active ingredients contained in Compound-Xueshuantong Capsule have the effect of resisting the neovascularization, retinal edema, inflammatory reaction, and oxidative stress injury caused by diabetic retinopathy. Therefore, the analysis shows that the compound Xuetong capsule may be more suitable for PDR with the main manifestation of neovascularization and NPDR with macular edema. However, these findings need to be confirmed by further clinical studies. In addition, the combination of active components of the Compound-Xueshuantong Capsule in diabetic retinopathy treatment may be the basis for optimizing new drugs for the treatment of diabetic retinopathy.

## Figures and Tables

**Table 1:** The top 10 active components in Compound-Xueshuantong Capsule.

Traditional Chinese medicine	Component	Degree	Betweenness centrality
HuangQi, SanQi	Quercetin	160	0.17766737
DanShen	Luteolin	34	0.04646771
HuangQi	Kaempferol	34	0.04183024
SanQi, XuanShen	Beta-sitosterol	24	0.00928675
HuangQi	Isorhamnetin	14	0.00976904
DanShen	Tanshinone IIa	12	0.01089857
DanShen	Cryptotanshinone	10	0.00746128
HuangQi	Formononetin	10	0.00126356
DanShen	Dehydrotanshinone II A	8	0.00126356
DanShen	Dan-shexinkum d	8	0.00126356

**Table 2:** Biological process of Compound-Xueshuantong Capsule in diabetic retinopathy treatment.

ID	Description	Gene ID	Count
GO: 0004879	Nuclear receptor activity	PGR/ESR1/AR/PPARG/NR3C1/RXRB/AHR	7
GO: 0098531	Transcription factor activity, direct ligand regulated sequence-specific DNA binding	PGR/ESR1/AR/PPARG/NR3C1/RXRB/AHR	7
GO: 0003707	Steroid hormone receptor activity	PGR/NR3C2/ESR1/AR/PPARG/NR3C1/RXRB	7
GO: 0001228	Transcription activator activity, RNA polymerase II-specific	PGR/ESR1/AR/RELA/NR3C1/FOS/RXRB/HIF1A/NFE2L2/PARP1/IRF1	11
GO: 0097153	Cysteine-type endopeptidase activity involved in apoptotic process	CASP9/CASP3/CASP7/CASP1	4
GO: 0005496	Steroid binding	PGR/NR3C2/ESR1/AR/NR3C1/CAV1	6
GO: 0001085	RNA polymerase II transcription factor binding	ESR1/AR/PPARG/FOS/AHR/NFE2L2	6
GO: 0030331	Estrogen receptor binding	ESR1/PPARG/PCNA/PARP1	4
GO: 0001221	Transcription cofactor binding	ESR1/RELA/AHR/NFE2L2	4
GO: 0042826	Histone deacetylase binding	RELA/CCND1/MAPK8/HIF1A/PARP1	5
GO: 0001091	RNA polymerase II basal transcription factor binding	ESR1/AR/AHR	3
GO: 0001223	Transcription coactivator binding	ESR1/RELA/AHR	3
GO: 0005178	Integrin binding	EGFR/ICAM1/VCAM1/PRKCA/IGF2	5
GO: 0035257	Nuclear hormone receptor binding	ESR1/PPARG/PCNA/HIF1A/PARP1	5
GO: 0051117	ATPase binding	PGR/ESR1/AR/CAV1	4
GO: 0033613	Activating transcription factor binding	PPARG/RELA/FOS/NFE2L2	4
GO: 0035258	Steroid hormone receptor binding	ESR1/PPARG/PCNA/PARP1	4
GO: 0016504	Peptidase activator activity	APP/CAV1/CASP1	3
GO: 0051427	Hormone receptor binding	ESR1/PPARG/PCNA/HIF1A/PARP1	5
GO: 0042562	Hormone binding	ACHE/AR/EGFR/NR3C1	4

**Table 3:** Target enrichment pathway of Compound-Xueshuantong Capsule in diabetic retinopathy treatment.

ID	Description	Gene ID	Count
hsa04933	AGE-RAGE signaling pathway in diabetic complications	RELA/VEGFA/CCND1/IL6/CASP3/ICAM1/BCL2/MAPK8/SELE/VCAM1/PRKCA/NOS3	12
hsa05418	Fluid shear stress and atherosclerosis	RELA/VEGFA/ICAM1/GSTP1/BCL2/FOS/MAPK8/SELE/VCAM1/GSTM1/CAV1/NOS3/NFE2L2	13
hsa04210	Apoptosis	RELA/CASP9/CASP3/CASP7/MCL1/BIRC5/BCL2/FOS/MAPK8/RAF1/PARP1/CTSD	12
hsa04668	TNF signaling pathway	RELA/IL6/CASP3/CASP7/ICAM1/FOS/MAPK8/SELE/VCAM1/IRF1	10
hsa05210	Colorectal cancer	EGFR/CCND1/CASP9/CASP3/BIRC5/BCL2/FOS/MAPK8/RAF1	9
hsa05161	Hepatitis B	RELA/CASP9/IL6/CASP3/PCNA/BIRC5/BCL2/FOS/MAPK8/RAF1/PRKCA	11
hsa05215	Prostate cancer	AR/RELA/EGFR/CCND1/CASP9/GSTP1/BCL2/PLAU/RAF1	9
hsa05167	Kaposi sarcoma-associated herpesvirus infection	RELA/VEGFA/CCND1/CASP9/IL6/CASP3/ICAM1/FOS/MAPK8/RAF1/HIF1A	11
hsa05133	Pertussis	RELA/IL6/CASP3/CASP7/FOS/MAPK8/IRF1/CASP1	8
hsa04215	Apoptosis-multiple species	CASP9/CASP3/CASP7/BIRC5/BCL2/MAPK8	6
hsa05205	Proteoglycans in cancer	ESR1/EGFR/VEGFA/CCND1/CASP3/PLAU/RAF1/PRKCA/HIF1A/CAV1/IGF2	11
hsa04917	Prolactin signaling pathway	ESR1/RELA/CCND1/FOS/MAPK8/RAF1/IRF1	7
hsa04066	HIF-1 signaling pathway	RELA/EGFR/VEGFA/IL6/BCL2/PRKCA/HIF1A/NOS3	8
hsa05212	Pancreatic cancer	RELA/EGFR/VEGFA/CCND1/CASP9/MAPK8/RAF1	7
hsa05163	Human cytomegalovirus infection	RELA/EGFR/VEGFA/CCND1/CASP9/IL6/CASP3/RAF1/PRKCA/PTGER3	10
hsa04926	Relaxin signaling pathway	RELA/CASP9/IL6/CASP3/CASP7/CASP1	6
hsa05134	Legionellosis	RELA/CASP9/IL6/CASP3/NFKBIA/CASP7/CASP8/HSF1/CASP1	9
hsa04370	VEGF signaling pathway	VEGFA/CASP9/RAF1/PRKCA/NOS3/HSPB1	6
hsa04151	PI3K-Akt signaling pathway	RELA/EGFR/VEGFA/CCND1/CASP9/IL6/MCL1/BCL2/RAF1/PRKCA/NOS3/IGF2	12
hsa04915	Estrogen signaling pathway	PGR/ESR1/EGFR/BCL2/FOS/RAF1/NOS3/CTSD	8

## Data Availability

The data used to support the findings of this study are available from the first author upon request.
